# Prevalence and Molecular Characterization of Second-Line Drugs Resistance among Multidrug-Resistant* Mycobacterium tuberculosis* Isolates in Southwest of China

**DOI:** 10.1155/2017/4563826

**Published:** 2017-07-17

**Authors:** Y. Hu, L. Xu, Y. L. He, Y. Pang, N. Lu, J. Liu, J. Shen, D. M. Zhu, X. Feng, Y. W. Wang, C. Yang

**Affiliations:** ^1^Tuberculosis Reference Laboratory, Chongqing Tuberculosis Control Institute, Chongqing 400050, China; ^2^Department of Pathogenic Organisms, Chongqing Medical University, Chongqing 400016, China; ^3^Molecular Medicine and Cancer Research Center, Chongqing Medical University, Chongqing 400016, China; ^4^National Tuberculosis Reference Laboratory, National Center for TB Control and Prevention, Chinese Center for Disease Control and Prevention, Beijing 102206, China; ^5^Chongqing Hospital of Traditional Chinese Medicine, Chongqing 400021, China

## Abstract

This study aimed to investigate the prevalence of multidrug-resistant tuberculosis (MDR-TB) isolates resistant to the second-line antituberculosis drugs (SLDs) and its association with resistant-related gene mutations in* Mycobacterium tuberculosis* (*M.tb*) isolates from Southwest of China. There were 81 isolates resistant to at least one of the SLDs among 156 MDR-TB isolates (81/156, 51.9%). The rates of general resistance to each of the drugs were as follows: OFX (66/156, 42.3%), KAN (26/156, 16.7%), CAP (13/156, 8.3%), PTO (11/156, 7.1%), PAS (22/156, 14.1%), and AMK (20/156, 12.8%). Therefore, the most predominant pattern was resistant to OFX compared with other SLDs (*P* < 0.001). The results of sequencing showed that 80.2% OFX-resistant MDR-TB isolates contained* gyrA* mutation and 88.5% KAN-resistant isolates had* rrs* mutations with the most frequent mutation being A1401G. These results suggest that improper use of SLDs especially OFX is a real threat to effective MDR-TB treatment not only in China but also in the whole world. Furthermore the tuberculosis control agencies should carry out SLDs susceptibility testing and rapid screening in a broader population of TB patients immediately and the SLDs should be strictly regulated by the administration in order to maintain their efficacy to treat MDR-TB.

## 1. Introduction

Tuberculosis (TB) remains a public threat to human health around the whole world; WHO estimates that, in 2015, 10.4 million people became ill with active TB and 1.4 million died from the disease, making it the world's leading cause of death by a single infectious agent [1]. In China, there are about 1.1 to 1.5 million new TB cases per year [2]. During the 1990s, multidrug-resistant tuberculosis (MDR-TB), defined as resistant to at least isoniazid and rifampin, emerged as a threat to TB control [3, 4]. Globally, 480,000 new TB cases (3.9%) are MDR-TB in 2015 [1]. In China, the proportion of MDR-TB cases is growing dramatically, and national survey estimated that there were 110,000 cases of MDR-TB and 8,200 cases of XDR-TB [5]. MDR-TB treatment requires the use of second-line drugs (SLDs) that are less effective, more toxic, and costlier than first-line isoniazid- and rifampin-based regimens. Recent studies have suggested that resistance to SLDs has arisen as a new threat [6–8], leading to extensively drug-resistant tuberculosis (XDR-TB), which has been found in 117 countries/regions thus far [1], and even so-called totally drug-resistant tuberculosis [6, 9].

There is a high incidence of TB in the Southwest of China. From 2014, local tuberculosis control agencies started the investigation of resistance to SLDs in MDR-TB isolates. Therefore this is the first report about the distribution characteristics of SLDs-resistant MDR-TB isolates and the main molecular mechanisms of SLDs resistance in this region. These results will provide a reference and basis for the development of rational MDR-TB chemotherapy regimen and the experience of MDR-TB control strategy inside and outside this region.

## 2. Materials and Methods

*Ethical Approval*. This study was approved by the Ethics Committee of the Chongqing Tuberculosis Control Institute. Patients enrolled in this study were given a subject information sheet, and all gave written informed consent to participate in this study.

*Patient Enrollment.* 677 smear-positive tuberculosis patients who were registered at local tuberculosis control agencies between January 2015 and August 2016 from 39 Districts of Chongqing Municipality, China, were enrolled in this study. Demographic information was obtained by review of medical record. The isolates were transferred to Chongqing Tuberculosis Control Institute for further drug susceptibility testing. According to the drug treatment history, the MDR-TB cases were divided into 4 groups: the previously treated cases, previously treated failure cases, retreatment cases, and retreatment failure cases, based on the China TB control program implementation guide (2008). The previously treated cases represent the pulmonary tuberculosis patients, still sputum smear-positive at the end of 3-month treatment. The previously treated failure cases represent the previously pulmonary tuberculosis patients, still sputum smear-positive after the whole treatment (6 months) or treatment for 5 months. The retreatment cases are defined as pulmonary tuberculosis patients, who were once cured but relapsed or were treated more than 1 month but treatment interruption was longer than 2 months. And the retreatment failure cases are defined as retreated pulmonary tuberculosis patients, still sputum smear-positive after the whole treatment (9 months) or treatment for 5 months.

*Drug Susceptibility Testing.* Drug susceptibility testing (DST) was performed using the Löwenstein-Jensen (L-J) proportion method (PM) [10]. The concentrations of drugs in L-J medium were as follows: 0.2 *μ*g/ml for isoniazid (INH), 40 *μ*g/ml for rifampicin (RIF), 2 *μ*g/ml for ofloxacin (OFX), 30 *μ*g/ml for kanamycin (KAN), 40 *μ*g/ml for capreomycin (CAP), 40 *μ*g/ml for prothionamide (PTO), 1 *μ*g/ml for p-aminosalicylic acid (PAS), and 30 *μ*g/ml for amikacin (AMK) [11, 12]. Results were read 28 days after inoculation and the reference* M.tb* H37Rv strain was used as a control. A strain was declared resistant to an antimicrobial agent when the growth rate exceeded 1% compared with the control. The MDR-TB strains were defined as those resistant to both isoniazid and rifampicin. In addition, Pre-XDR-TB is defined as MDR-TB with additional resistance to either a fluoroquinolone (FQ) or an injectable (kanamycin, amikacin, or capreomycin), but not to both a FQ and an injectable, and XDR-TB resistant to any fluoroquinolone, and at least one of three second-line injectable drugs (capreomycin, kanamycin, and amikacin), in addition to MDR [11, 12].

*DNA Extraction and Amplification*. The colonies from the surface of L-J medium were suspended in 500 *μ*l Tris-EDTA (TE) buffer and heated in a 95°C water bath for 1 h. Genomic gene was extracted by a conventional method [13]. The DNA was used as template for amplification and the primers were shown in Table 1. The 50 *μ*L PCR mixture was prepared as follows: 25 *μ*l, 2x GoldStar MasterMix (CWBio, Beijing, China), 5 *μ*L DNA template, and 0.2 *μ*M of each primer set. PCR parameters for amplification were 5 min at 94°C, followed by 35 cycles at 94°C for 1 min, 58°C for 1 min, 72°C for 1 min, and a final extension at 72°C for 10 min. PCR products were sent to Tsingke company for sequencing. All sequences were aligned with* gyrA*,* gyrB, rrs, *and* eis* of the reference strain H37Rv (ATCC27294) using BioEdit (version 7.1.3.0) software.

*Statistical Analysis*. Chi square test was used to evaluate the associations among multiple categorical variables, and the statistical results were summarized with odds ratios (ORs) with 95% confidence intervals (CIs). And Fisher's exact test was used if any expected counts are less than 5. All calculations were performed in SPSS 13.0 (SPSS Inc., USA).

## 3. Results

*Demographic Characteristics and Drug Susceptibility Profiles*. A total of 156 (23.0%) of 677 clinical isolates were identified as MDR-TB, including 53 (34.0%) pre-XDR and 20 (12.8%) XDR. Overall, 121 (77.6%) strains were isolated from male patients and 35 (22.4%) from female patients. The average age of the patients was 47.5 years old (range 15–80 years), and the patients in 60–69 years old group had a significantly lower risk of resistance to the SLDs than those in other groups (OR, 4.533; 95%CI, 1.303 to 15.772; *P* = 0.014) (Table 2). In addition, 79 (50.6%) of isolates were from urban areas (within an hour drive from the downtown) of Chongqing and 77 (49.4%) were from suburb. The retreated case were 85 (54.5%) which occupied the large proportion. Compared with the previously treated failure group, the retreatment failure group had greater risk of second-line-drug resistance (*P* < 0.05) (Table 2).

**Figure 1:**
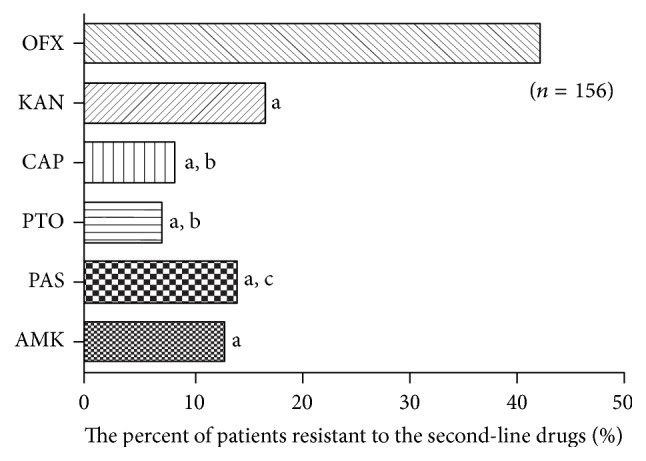
Distribution of MDR isolates resistant to different second-line drugs. ^a^Compared with OFX group, *P* < 0.001. ^b^Compared with KAN group, *P* < 0.05. ^c^Compared with PTO group, *P* < 0.05.

*Second-Line Anti-TB Drug-Resistant Pattern*.  Table 3 described the distribution of the 156 MDR isolates according to second-line-drug-resistant patterns. We identified 2 groups of MDR isolates: (i) those with resistance to one of the SLDs included OFX, KAN, CAP, PTO, PAS, and AMK (monoresistant) and (ii) those with resistance to more than one of the SLDs mentioned above (multiresistant). And there were 81 isolates resistant to at least one of the six second-line anti-TB drugs among 156 MDR isolates. The most predominant pattern was monoresistant to OFX (34/156, 21.79%). Combining the number of monoresistant and multiresistant isolates and calculating their percentages with respect to the 156 MDR-TB isolates, the rates of general resistance to each of the drugs were as follows: OFX (66/156, 42.3%), KAN (26/156, 16.7%), CAP (13/156, 8.3%), PTO (11/156, 7.1%), PAS (22/156, 14.1%), and AMK (20/156, 12.8%) (Figure 1). Furthermore, compared with KAN, CAP, PTO, PAS, or AMK, OFX is more likely to be the second-line drug resistance by the MDR isolates (*P* < 0.001). In addition, comparing the resistance rate of KAN with CAP or PTO, as well as PTO with PAS, also had significant difference (*P* < 0.05) (Figure 1).

*Risk Factors for the Pre-XDR-TB/XDR-TB from 156 MDR Isolates from Southwest of China*. As expected, the age and the district maybe independent factors associated with Pre-XDR-TB/XDR-TB isolates. Patients whose age is between 60 and 69 had a significantly lower risk of being a Pre-XDR-TB or XDR-TB cases than those at other ages (OR, 4.554; 95% CI, 1.228 to 16.881; *P* = 0.037) (Table 4).

*OFX Resistance with gyrA or gyrB Mutation*. Because known mechanisms of FQ resistance are caused by mutations in the quinolone resistant-determining region (QRDR) of the gene encoding subunit A or B of DNA gyrase (*gyrA* or* gyrB*), fragments comprising these regions were analyzed in this study. Among 156 MDR-TB isolates, 66 were resistant to OFX. Of them, 53 isolates (80.2%) contained mutations within* gyrA* (Table 5). The most predominant mutations occurred at codon 94 (34 isolates, 64.2%), where the Asp codon was replaced with a Gly (15 isolates, 28.3%), Ala (12 isolates, 22.6%), Tyr (1 isolates, 1.9%), or Asn (6 isolates, 11.3%) codon. The Asp94 mutations were also found in four combinations: one with an Ala74Ser mutation and three with an Ala90Val mutation. The Ala90Val mutation was the secondly predominant mutation (16 isolates, 30.2%). The Ala90 mutations were also found in three combinations: one with Gly88Ala mutation, one with an Asp94Gly mutation, and two with Asp94Ala mutation. Other mutations within* gyrA* included Gly88Cys (*n* = 1), Asp89Asn (*n* = 1), and Ser91Pro (*n* = 3). Mutations within* gyrB* were observed in four isolates (6.1%), with Asp461Asn (*n* = 1), and Gly512Arg (*n* = 3). Nine strains of OFX-susceptible isolates also display mutations within these two target fragments. Among them, the mutations within* gyrA* included Gly88Ala (*n* = 1), Ala90Val (*n* = 2), and Asp94Ala (*n* = 2) and mutations within* gyrB* were observed in four isolates with Asp461Asn (*n* = 3), and Gly512Arg (*n* = 1) (Table 5).

*KAN Resistance with eis or rrs Mutations*. Since resistance to KAN is caused by mutations in* rrs* or the promoter of* eis*, a 516-bp region of the open reading frame of* rrs* and the promoter of* eis* were investigated. Among all 156 MDR-TB isolates, 26 isolates were resistant to KAN, and 19 (88.5%) of these contained mutations within the* rrs* and 4 (15.4%) within* eis* region of interest, respectively (Table 6). None of the isolates contained mutations within both loci. The most frequent mutation of* rrs* region was A1401G, which was observed in 18 isolates (69.2%), as a single mutation. The other mutation within* rrs* region was G1339A (*n* = 1). Mutations within the promoter region of* eis* included the G(-10)A (*n* = 3) and C(-14)T (*n* = 1). We also found two KAN-susceptible MDR isolates harboring a A(1449)G mutation in* rrs* region and a G(-10)A mutation in the promoter of* eis,* respectively (Table 5).

*Association of Gene Mutation and Drug Resistance among 156 MDR-TB Isolates*. Table 6 showed that the isolates with* gyrA* gene mutation were closely related to the drug resistance to OFX (*P* < 0.001). In contrast, there was no significant difference in the proportion of isolates with mutations in* gyrB* gene between OFX-resistant and OFX-susceptible isolates. In addition, the isolates with* rrs* or* eis* gene mutation also had something to do with the drug resistance to KAN (*P* < 0.001, *P* = 0.003, resp.).

## 4. Discussion

TB is an underappreciated public health threat in developed nations. In 2015, an estimated 10.4 million new TB cases and 1.4 million deaths occurred worldwide; and 3.9% of these new cases were multidrug-resistant tuberculosis (MDR-TB) and extensively drug-resistant tuberculosis (XDR-TB) strains [1]. Drug resistance is a severe challenge to tuberculosis control, as it raises the possibility of a condition that can no longer effectively be treated with antituberculosis drugs and further transmission to public population [14]. This situation of MDR-TB highlights the urgent need for rapid and accurate drug susceptibility testing (DST) to optimize the treatment regimen and reduce the risk of acquired resistance [15]. Despite China having the second highest incidence of MDR-TB, information regarding MDR-TB and resistance to second-line antituberculosis drugs (SLDs) among MDR-TB isolates still remains unclear for many regions of China. This study is the first to detect SLDs resistance among MDR-TB isolates in Chongqing, Which is a very important city in Southwest of China.

Because of the diversity of TB epidemical situation, the actual use of SLDs, and other factors, the rate of SLDs resistance would be distinctive in different regions. This study showed that 51.9% of MDR-TB patients in Chongqing had resistance to at least one SLDs, which is slightly lower than Shanghai (54.4%) [16], another city of China, even worse than that in other reports, India (44.8%) [17], Russia (43.3%) [18], and Poland (30.4%) [19]. This indicated that the situation of SLDs resistance in this region is serious.

The results showed that the retreatment failure group had greater risk of SLDs resistance than previously treated failure group. As the patients in the retreatment failure group with a longer history of SLDs treatment were more likely to have isolates resistant to the drugs. This association, to some degree, could reflect the poor administration of drugs in health facilities, where some SLDs, such as fluoroquinolones (FQs), are easily and extensively prescribed for respiratory infections and other bacterial infections and in some cases even available without a prescription in local drug stories. Easy access and inappropriate use of these drugs increase the risk for the emergence of drug-resistant TB. Meanwhile our results implied the rate of resistance to OFX (42.3%) in MDR-TB isolates is highest among the six SLDs. This may be due to OFX as one of the main FQs has been extensively used for TB or other disease and the transmission of OFX-resistant* M*.*tb* directly in China; therefore this should be considered whether it is an optimal regimen for MDR-TB treatment in China.

Generally speaking, the demographic characteristics including the medical conditions and socioeconomic factors were related to the occurrence of tuberculosis resistance [5, 20]. Chongqing is located in Southwest of China and contains 39 districts and counties, of which 14 state-level poverty-stricken areas are in the “suburbs,” and has a high incidence of tuberculosis. However, this result showed that there was no statistical difference of SLDs-resistant MDR-TB isolates between urban areas and suburb. Maybe with the implementation of urban and rural development strategy of Chongqing, traffic is more convenient and population mobility is greater, so the occurrence of TB within the city is likely to spread. And reports revealed that SLDs-resistant TB arises mainly from direct transmission [16, 20–23]. On the other side, XDR-TB isolates in our study were significantly more in suburb (5.1%, 4/79) than those in urban areas (20.8%, 16/77) (*P* = 0.004). The main reason for this is that the people living in the suburb usually have poorer economic level, education level, and medical level than those in the urban areas. Therefore they also have inadequate knowledge of TB so that they have poor compliance of treatment and high rates of irregular medication.

An interesting finding of our study is that patients 60–69 years old had a significantly lower risk of SLDs resistance (*P* < 0.05) and also had a lower rate of Pre-XDR-TB/XDR-TB (*P* < 0.05) than those of other age. This result is consistent with another report of China [24] and a conclusion of review from European studies [25]. Although the exact reason is unknown, we hypothesize that the increased risk of harboring SLD resistance in patients under 60 years may be due to the previously exposure of SLDs, such as OFX which was uesd for anti-TB treatment from around 1985 in Chongqing; TB cases in older patients are usually considered as relapse cases, and the infecting strains may be more ancient and carry a lower risk of becoming resistant to OFX, which is associated with the emergence of resistance to SLDs among this special population.

OFX as one of FQs plays an important role in the treatment of various types of drug-resistant TB. FQs mainly act on the DNA gyrase in order to interfere DNA replication leading to bacterial death. The DNA gyrase is a tetramer composed of two A and two B subunits which are encoded by the* gyrA* and* gyrB* genes, respectively. The quinolone resistant-determining region (QRDR) is comprised of conserved area within* gyrA* and* gyrB* gene [26, 27]. It has been reported that mutations of* gyrA* gene in* M.tb *are closely related to quinolone resistance, while the mutations of* gyrB* gene are seldom related to the drug resistance [26–29]. Our results are consistent with these researches. The isolates of MDR-TB with mutations of* gyrA* gene were closely related to the drug resistance to OFX (*P* < 0.001). As comparison there were 90 cases which were selected from 521 non-MDR-TB cases randomly (data not shown), among which there were 84 OFX-susceptible strains and only one strain with the mutation of* gyrA* gene (1.2%); 6 OFX-resistant strains also contain one strain with mutation of* gyrA* gene (16.7%). In contrast, no significant correlation between OFX resistance and* gyrB* polymorphism was observed in this study. Hence, detection of the mutation of* gyrA* gene could be used to predict OFX resistance in Chongqing. Notably, there were 13 OFX-resistant strains without mutation located in* gyrA*, indicating that there are other mechanisms related to OFX resistance, such as cell wall permeability and drug efflux pump [30, 31]. On the other hand, there were 5 OFX-susceptible strains with mutation of* gyrA* gene, including one strain with an Gly88Ala mutation, two strains with Ala90Val mutation, and two strains with Asp94Ala mutation, respectively. These mutant types may be associated with low-level OFX resistance, which will be evaluated by MIC method in the future.

KAN is another one of important antituberculosis SLDs. It plays a role in inhibiting protein synthesis by 16SrRNA. And 16SrRNA is encoded by* rrs* gene whose mutation is currently known as the main mechanism of KAN resistance. Among them, the 1401 A → G point mutation is considered to be an important symbol of high-level resistance to KAN [32, 33]. Our observation is in agreement with these reports; 26 isolates were resistant to KAN, and 19 of these (73.1%) contained mutations within the* rrs* region. The most common mutation of* rrs* gene was A1401G, which was observed in 18 isolates (69.2%), as a single mutation. Interestingly, there was one KAN-resistant strain harboring G1339A substitution located in* rrs *region, which may serve as another mechanism contributing to KAN resistance. Besides* rrs*, the mutations in the promoter region of* eis* gene were associated with low-level resistance to KAN [34]. The most common mutation points in* eis* gene reported were the G(-10)A and C(-14)T. Our study supports these findings, as 4 isolates were found to have mutations in* eis *gene, including three of G(-10)A and one of C(-14)T. Thus, among 26 KAN-resistant isolates, 20 isolates (76.9%) contained mutations in either* rrs* or (and)* eis gene*, and 3 isolates contained mutations in both of the genes. This suggested that the sequencing of* rrs* and* eis* region could be a rapid filter method for KAN resistance before the DST results. And combining detection of the two regions might improve the sensitivity and accuracy of the drug resistance to KAN.

There were several limitations of this study. First, the present study only enrolled the previously treated TB patients rather than new cases due to the limitation in cost and human resource. Thus, we could not describe the drug-resistant profiles against SLDs among this population. Second, although a large number of clinical MDR-TB isolates were tested (*n* = 156), the number of SLDs-resistant isolates, especially OFX- resistant (*n* = 66) and KAN-resistant (*n* = 26) isolates, was still small. This might limit the detection of the variety of gene mutations. Third, there was still some drug-resistant isolates that could not be explained in our study, such as mutations at other gene regions [35], permeability of cell membrane, and overexpression of drug efflux pump gene [30, 31]. Therefore the other mechanisms of OFX resistance or KAN resistance will be detected in the further study and whole-genome sequencing might be needed to find more loci associated with drug resistance and to improve the performance of the sequencing-based assay.

In conclusion, our data indicated that 51.9% MDR-TB patients in Southwest of China were resistant to at least one of SLDs, and most of which were resistant to OFX (42.3%) and KAN (16.7%). The mutation of* gyrA* gene in* M.tb* is mainly mechanisms of OFX resistance. In addition, the mutation of* rrs* gene and the promoter of* eis* gene also have a close relationship with KAN resistance. Therefore the improper use of SLDs especially OFX for other infections is a real threat to effective MDR-TB treatment and OFX may be not an optimal regimen for MDR-TB patients. At present in China, the commercial drug-resistant test kits for screening target gene mutation are mainly on the first-line anti-TB drugs. However, the detection of gene mutations associated with the SLDs resistance has not yet been made universal. In addition to gene sequencing, the development of commercial kits for rapid molecular diagnosis of the SLDs-resistant-related genes is urgently needed to assist in controlling these severe forms of MDR-TB.

## Figures and Tables

**Table 1:** The Primers used for PCR.

Symbol	Gene ID	Primers	PCR	Tm
			Product length	
*gyrA*	887105	F: 5′-TCGACTATGCGATGAGCGTG-3′	415 bp	58°C
		R: 5′-GGTAGCACCGTCGGCTCTTG-3′		
*gyrB*	887081	F: 5′-GAGTTGGTGCGGCGTAAGAGC-3′	322 bp	60°C
		R: 5′-CGGCCATCAAGCACGATCTTG-3′		
*rrs*	2700429	F: 5′-GAGTTGGTGCGGCGTAAGAGC-3′	464 bp	58°C
		R: 5′-GGGGCGTTTTCGTGGTGCTCC-3′		
*eis*	885903	F: 5′-GCGTAACGTCACGGCGAAATTC-3′	567 bp	60°C
		R: 5′-GTCAGCTCATGCAAGGTG-3′		

**Table 2:** Risk factors of second-line drug resistance among 156 MDR^a^ isolates.

Characteristics	Number (%) of isolates (*n* = 156)	Number (%) of isolates		OR (95% CI)	*P*
		Resistant^b^	Susceptible^c^		
Sex					
Female	35 (22.4)	21 (25.9)	14 (18.6)	—	—
Male	121 (77.6)	60 (74.1)	61 (81.3)	1.356 (0.635–2.894)	0.431
Age					
~29	29 (18.6)	17 (21.0)	12 (16.0)	—	—
30~39	33 (21.2)	19 (23.5)	14 (18.7)	1.044 (0.380–2.870)	0.934
40~49	40 (25.6)	19 (23.5)	21 (28.0)	1.566 (0.597–4.110)	0.361
50~59	25 (16.0)	16 (19.8)	9 (12.0)	0.797 (0.265–2.397)	0.686
60~69	21 (13.5)	5 (6.2)	16 (21.3)	4.533 (1.303–15.772)	0.014
≧70	8 (5.1)	5 (6.2)	3 (4.0)	0.850 (0.170–4.256)	1.000^h^
District					
Urban	79 (50.6)	36 (44.4)	43 (57.3)	—	—
Suburb	77 (49.4)	45 (55.6)	32 (42.7)	0.595 (0.316–1.122)	0.108
Treatment history					
Previously treated failure cases^d^	18 (11.5)	6 (7.4)	12 (16.0)	—	—
Previously treated cases^e^	30 (19.2)	18 (22.2)	12 (16.0)	0.333 (0.098–1.132)	0.074
Retreatment failure cases^f^	23 (14.7)	15 (18.5)	8 (10.7)	0.267 (0.072–0.9814)	0.043
Retreatment cases^g^	85 (54.5)	42 (51.9)	43 (57.3)	0.512 (0.176–1.490)	0.214

^a^MDR is defined as *Mycobacterium tuberculosis* strain resistant to at least isoniazid and rifampin. ^b^The isolate strain is resistant to one of the second-line drugs as follows: OFX, KAN, AMK, CAP, PTO, or PAS; ^c^The isolate strain is susceptible to all of the second-line drugs as follows: OFX, KAN, AMK, CAP, PTO, and PAS. ^d^Previously treated failure cases, still sputum smear-positive after the whole treatment (6 months) or treatment for 5 months. ^e^Previously treated cases, still sputum smear-positive at the end of 3-month treatment. ^f^Retreatment failure case, still sputum smear-positive after the whole treatment (9 months) or treatment for 5 months. ^g^Retreatment cases, who were once cured but relapsed or were treated more than 1 month but treatment interruption was longer than 2 months; now accept treatment again; ^h^*P* value from Fisher's exact test.

**Table 3:** Patterns of drug resistance to second-line^a^ drugs among 156 MDR^b^ isolates from Southwest of China.

Patterns	Number of isolates (*n* = 156)	Number (%)
Monoresistant	43	27.56
OFX	34	21.79
KAN	1	0.64
PTO	2	1.28
PAS	6	3.85
Multiresistant	38	24.36
OFX + KAN	3	1.92
OFX + KAN + AMK	2	1.28
OFX + KAN + CAP	1	0.64
OFX + KAN + PAS	1	0.64
OFX + KAN + CAP + AMK	4	2.56
OFX + KAN + PTO + AMK	1	0.64
OFX + KAN + PTO + PAS + AMK	1	0.64
OFX + KAN + CAP + PAS + AMK	5	3.21
OFX + KAN + CAP + PTO + PAS + AMK	1	0.64
OFX + AMK	1	0.64
OFX + PTO	5	3.21
OFX + PAS	7	4.49
KAN + AMK	2	1.28
KAN + CAP + AMK	2	1.28
KAN + PTO + AMK	1	0.64
KAN + PAS	1	0.64
Susceptible	75	48.08

^a^The second-line drugs are as follows: OFX, KAN, AMK, CAP, PTO, or PAS; ^b^MDR is defined as *Mycobacterium tuberculosis* strains resistant to at least isoniazid and rifampin.

**Table 4:** The effect of age on the Pre-XDR/XDR isolates from Southwest of China.

Age	Number (%) of isolates (*n* = 156)	Number (%) of Pre-XDR/XDR isolates (*n* = 73)	OR (95% CI)	*P*
~29	29 (18.6)	15 (20.5)	—	—
30~39	33 (21.2)	18 (24.7)	0.893 (0.328–2.427)	0.824
40~49	40 (25.6)	17 (23.3)	1.450 (0.554–3.790)	0.446
50~59	25 (16.0)	14 (19.2)	0.842 (0.288–2.465)	0.753
60~69	21 (13.5)	4 (5.5)	4.554 (1.228–16.881)	0.037^a^
≧70	8 (5.1)	5 (6.8)	0.643 (0.128–3.203)	0.588

^a^ *P* value from Fisher's exact test.

**Table 5:** The characteristics of *gyrA* and *gyrB *gene mutations in 156 MDR isolates.

(Number of isolates)	Locus	Codon/nucleotide change(s)	Amino acid^b^/nucleotide change(s)	Number of strains	Number (%)
OFX-resistant (66)	*gyrA*	NM	NM	13	19.7
		GGC→TGC	Gly88Cys	1	1.5
		GAC→AAC	Asp89Asn	1	1.5
		GCG→GTG	Ala90Val	13	19.7
		TCG→CCG	Ser91Pro	3	4.5
		GAC→AAC	Asp94Asn	6	9.1
		GAC→GCC	Asp94Ala	10	15.2
		GAC→GGC	Asp94Gly	13	19.7
		GAC→TAC	Asp94Tyr	1	1.5
		GCC→TCC/GAC→GGC	Ala74Ser/Asp94Gly	1	1.5
		GCG→GTG/GAC→GCC	Ala90Val/Asp94Ala	2	3.0
		GCG→GTG/GAC→GGC	Ala90Val/Asp94Gly	1	1.5
		GGC→GCC/GCG→GTG	Gly88Ala/Ala90Val	1	1.5
	*gyrB*	NM	NM	62	93.9
		GAC→AAC	Asp461Asn	1	1.5
		GGG→AGG	Gly512Arg	3	4.5
OFX-susceptible (90)	*gyrA*	NM	NM	85	94.4
		GGC→GCC	Gly88Ala	1	1.1
		GCG→GTG	Ala90Val	2	2.2
		GAC→GCC	Asp94Ala	2	2.2
	*gyrB*	NM	NM	86	95.6
		GAC→AAC	Asp461Asn	3	3.3
		GGG→AGG	Gly512Arg	1	1.1
KAN-resistant (26)	*rrs*	NM	NM	7	26.9
		G→A	G1339A	1	3.8
		A→G	A1401G	18	69.2
	*eis*	NM	NM	22	84.6
		G→A	G(−10)A	3	11.5
		C→T	C(−14)T	1	3.8
KAN-susceptible (130)	*rrs*	NM	NM	129	99.2
		A→G	A(1449)G	1	0.8
	*eis*	NM	NM	129	99.2
		G→A	G(−10)A	1	0.8

NM, no mutation; ^b^Amino acid numbers are based on homologous mutations in* Escherichia coli*.

**Table 6:** The relationship between gene mutation and drug resistance in 156 MDR isolates.

Drug-resistant phenotype	Number of isolates	Locus	Number (%) of isolates		*χ* ^2^	*P*
			Mutation	Without mutation		
OFX		*gyrA*				
Resistant	66		53 (80.3)	13 (19.7)	91.086	<0.001
Susceptible	90		5 (5.6)	85 (94.4)		
		*gyrB*				
Resistant	66		4 (6.1)	62 (93.9)	0.007	0.932^a^
Susceptible	90		4 (4.4)	86 (95.6)		
KAN		*rrs*				
Resistant	26		19 (73.1)	7 (26.9)		<0.001^b^
Susceptible	130		1 (0.8)	129 (99.2)		
		*eis*				
Resistant	26		4 (15.4)	22 (84.6)		0.003^b^
Susceptible	130		1 (0.8)	129 (99.2)		

^a^Continity correction; ^b^Fisher's exact test.
